# Paraneoplastic Hepatitis Associated with Relapsed Nodular Lymphocyte-Predominant Hodgkin Lymphoma

**DOI:** 10.3390/hematolrep18020018

**Published:** 2026-02-28

**Authors:** Jasmin Nelissen, Sandra Coenen, King Lam, Michael Doukas, Harry L. A. Janssen, Yasmina Serroukh

**Affiliations:** 1 Department of Hematology, Erasmus Medical Center, 3015 GD Rotterdam, The Netherlands; y.serroukh@erasmusmc.nl; 2 Department of Gastroenterology and Hepatology, Erasmus Medical Center, 3015 GD Rotterdam, The Netherlands; s.coenen@erasmusmc.nl (S.C.); h.janssen@erasmusmc.nl (H.L.A.J.); 3 Department of Pathology, Erasmus Medical Center, 3015 GD Rotterdam, The Netherlands; k.lam@erasmusmc.nl (K.L.); m.doukas@erasmusmc.nl (M.D.)

**Keywords:** nodular lymphocyte-predominant Hodgkin lymphoma, paraneoplastic hepatitis, hepatic fibrosis

## Abstract

**Background**: Nodular lymphocyte-predominant Hodgkin lymphoma (NLPHL) is an indolent B-cell lymphoma with long-term survival and a tendency for late relapse. Hepatic manifestations of varying etiologies have been described in lymphoproliferative disorders. However, paraneoplastic hepatitis is rare, and reports typically describe acute presentations. We describe an unusual case of paraneoplastic hepatitis with an indolent and progressive clinical course occurring in the setting of relapsed NLPHL. **Case Presentation**: A 32-year-old man with a history of NLPHL was found to have marked transaminase elevation with preserved liver function during routine follow-up. Extensive evaluation excluded viral, autoimmune, and metabolic causes of liver disease. Liver biopsy demonstrated confluent and bridging necrosis with lymphoplasmacytic infiltrates, without evidence of direct lymphoma involvement. Excisional biopsy of a cervical lymph node revealed relapse of NLPHL without histologic transformation. Treatment with corticosteroids resulted in partial biochemical improvement, and subsequent rituximab monotherapy achieved lymphoma remission. Despite this, low-grade transaminase elevation persisted, and follow-up imaging and liver biopsy demonstrated progression to fibrosis, suggesting a tendency towards chronicity. **Conclusions**: Paraneoplastic hepatitis should be considered in patients with NLPHL who present with unexplained liver abnormalities. This report illustrates a fibrosing form of paraneoplastic hepatitis associated with NLPHL and broadens the clinical spectrum of paraneoplastic hepatic injury. Early recognition, histological confirmation, and tailored immunosuppressive management are critical to optimizing hepatic and lymphoma-related outcomes.

## 1. Introduction

Nodular lymphocyte-predominant Hodgkin lymphoma (NLPHL) is a rare, low-grade B-cell lymphoma that accounts for approximately 5% of all Hodgkin lymphoma (HL) cases. It is historically classified as a subtype of HL. However, the International Consensus Classification (ICC) recently classified this entity as “nodular lymphocyte-predominant B-cell lymphoma (NLPBL)” within the mature B-cell neoplasms. For now, the fifth edition of the World Health Organization (WHO) has kept the term NLPHL while in preparation of new nomenclature. In contrast to classic Hodgkin lymphoma (cHL), NLPHL does not present with Reed–Sternberg cells but is characterized by lymphocyte-predominant cells surrounded by reactive small B- and T-lymphocytes. NLPHL expresses mature B-cell markers, including CD20, CD79a, and BCL6, and lacks CD15 and CD30. This biologic distinction is reflected clinically, as NLPHL generally behaves as an indolent lymphoma. Patients typically present with early-stage disease and localized peripheral lymphadenopathy. Long-term survival outcomes are favorable despite a propensity for late relapse and occasional transformation to aggressive B-cell lymphoma. Extranodal involvement and systemic inflammatory manifestations are generally uncommon [1,2].

Lymphoproliferative disorders frequently present with hepatic abnormalities, ranging from mild elevations in liver function tests (LFTs) to fulminant hepatic failure. Primary hepatic lymphoma remains exceedingly rare, whereas direct lymphomatous infiltration or extrahepatic biliary obstruction are observed more often. In most cases, liver disturbances reflect systemic disease, such as viral infections, drug-induced liver disease, hemophagocytic syndrome, or immune-mediated processes [3]. Paraneoplastic hepatic dysfunction has been described in association with several lymphoma subtypes, but most reported cases occur in cHL. These syndromes are believed to result from immune-mediated mechanisms or toxic cytokines triggered by the underlying malignancy, leading to hepatic injury. Vanishing bile duct syndrome, characterized by intrahepatic bile duct loss and portal fibrosis secondary to cytokine release, is a well-recognized paraneoplastic manifestation of cHL [4]. Another paraneoplastic cause of cholestatic jaundice is idiopathic cholestasis without ductopenia, and it has been reported as a presenting symptom of cHL [5]. Paraneoplastic hepatitis in lymphoproliferative disorders without direct liver infiltration has been reported in only a small number of patients, including two cases of cHL [6,7] and one case of NLPHL [8].

Paraneoplastic syndromes (PNSs) in NLPHL are exceptionally rare. Based on the available literature, neurological manifestations appear to be the most frequently described PNS in NLPHL [8,9,10,11,12], similarly to cHL observations. In a recent systematic review by El Fakih et al. summarizing PNS in 128 patients with cHL, neurological manifestations were the most frequently reported, followed by hepatic pathology [13]. The higher incidence of paraneoplastic phenomena in cHL is thought to be related to loss of B-cell identity, the clonal presence of Epstein–Barr virus, and dysregulated immune signaling, all of which contribute to a pronounced inflammatory tumor microenvironment [14]. In contrast, NLPHL typically follows a more indolent clinical course and is associated with less systemic inflammatory activation, which may explain the lower propensity for immune-mediated paraneoplastic manifestations, including hepatic injury.

Whereas previous cases presented with acute liver failure, our report portrays a more indolent clinical course of paraneoplastic hepatitis as the presenting feature of relapsed NLPHL, which progressed insidiously to hepatic fibrosis despite complete remission of the underlying lymphoma.

## 2. Case Presentation

A 32-year-old male presented with asymptomatic elevation of LFTs during routine follow-up. He reported intermittent fatigue and epigastric pain but denied fever, night sweats, or weight loss. There were no recent changes in medication or high-risk infectious exposures. He did not use recreational drugs or herbal supplements. He did report having an episode of alcohol binge drinking the preceding weekend. Physical examination was unremarkable, with no hepatosplenomegaly or palpable lymphadenopathy. At age 21, he was diagnosed with NLPHL stage IA and treated with radiotherapy. At progression three years later, he achieved complete remission (CR) following six cycles of rituximab combined with ABVD (doxorubicin, bleomycin, vinblastine, and dacarbazine). He relapsed at age 29 and received four cycles of rituximab, achieving a partial response (PR). Liver function tests had remained normal throughout all previous treatments.

**Figure 1:**
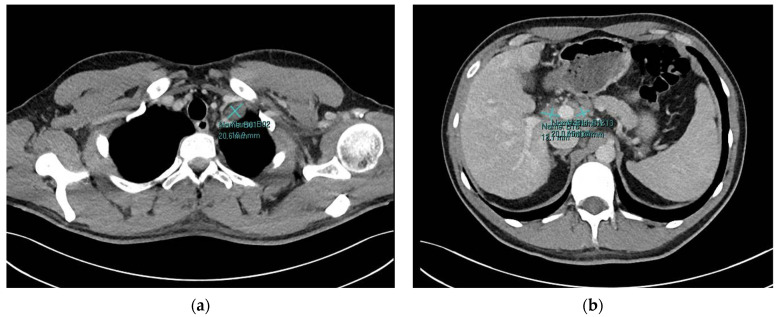
CT scan at relapse showing (**a**) a supraclavicular lymph node measuring 16 × 20 mm and (**b**) hepatic hilar lymph nodes measuring 13 × 20 mm and 15 × 10 mm.

**Figure 2:**
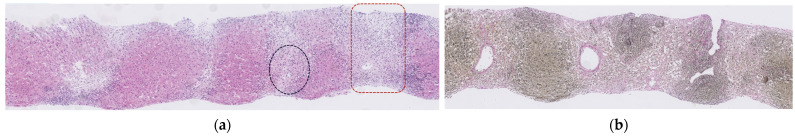
Liver biopsy at diagnosis: (**a**) HE stain (31× objective lens equivalent). Liver parenchyma with preserved architecture and features of confluent (black dotted circle) and bridging (red dotted square) necrosis; (**b**) collagen stain (elastin) without recognizable elastin fibers.

**Figure 3:**
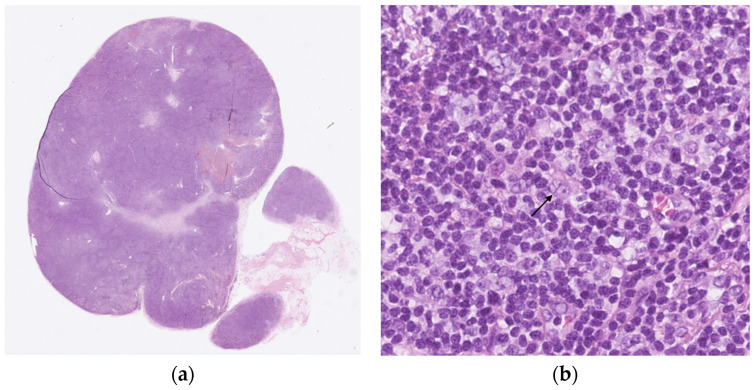
Lymph node biopsy at relapse: (**a**) lymphoid tissue with a vague nodular architecture composed predominantly of small lymphocytes and macrophages; (**b**) hematoxylin and eosin (HE) stain (40× objective lens equivalent). Lymphocyte-predominant cells (“popcorn cells”) with a multi-lobated nucleus and small nucleoli (arrow), characteristic of NLPHL.

At presentation, the patient was under active surveillance every 3 months and remained asymptomatic. Laboratory tests showed elevations in total bilirubin 1.8 mg/dL (<1), alanine transaminase (ALT) 1541 U/L (<45), aspartate transaminase (AST) 804 U/L (<35), gamma-glutamyl transferase (GGT) 211 U/L (<55), and lactate dehydrogenase (LDH) 370 U/L (<248). Liver synthetic function remained largely preserved, with an international normalized ratio (INR) of 1.2 and albumin of 43 g/L. Complete blood count was normal with hemoglobin 15.7 g/dL, white blood cell count 3.5 × 10^9^/L, and platelet count 144 × 10^9^/L. Viral workup for hepatitis A, B, C, and E and human immunodeficiency virus (HIV) was negative, as were cytomegalovirus (CMV) and Epstein–Barr virus (EBV) polymerase chain reactions. Autoimmune testing was negative for antinuclear autoantibodies, antimitochondrial antibodies, anti-smooth muscle, and anti-liver/kidney microsomal antibodies. There was no evidence for Wilson’s disease with normal ceruloplasmin or hemochromatosis with elevated ferritin attributed to inflammation. An abdominal ultrasound demonstrated mild hepatic steatosis without evidence of cirrhosis or focal hepatic lesions. Hepatopetal flow was observed in the portal vein, and the hepatic veins and hepatic artery were patent. The bile ducts, gallbladder, pancreas, and spleen (measuring 12.0 cm) appeared normal. Computed tomography (CT) of his neck, chest, abdomen, and pelvis (Figure 1) showed progression of lymphadenopathy, with enlargement of left supraclavicular lymph nodes measuring up to 20.6 × 16.0 mm (previously 12 × 11 mm) and 19.3 × 8.7 mm (previously 9 × 6 mm), as well as mild enlargement of additional cervical lymph nodes. Newly developed lymphadenopathy was identified at the hepatic hilum, with lymph nodes measuring 13.1 × 20.0 mm, 15.4 × 10.7 mm, and 18.4 × 14.3 mm. Furthermore, a portocaval lymph node measuring 24.1 × 11.0 mm was observed. Newly prominent but subcentimetric mediastinal lymph nodes were also noted. The liver demonstrated heterogeneous parenchymal enhancement without focal lesions. A mild increase in splenic size was observed, 11.5 cm (10.7 cm previously). The pancreas, adrenal glands, and kidneys appeared normal. Liver core needle biopsy (Figure 2) revealed mild to focally moderate portal and lobular hepatitis with interface activity and parenchymal nodularity, reflecting recent parenchymal collapse. There was hepatocellular loss in pericentral distribution with areas of confluent and bridging necrosis. The portal tracts showed inflammatory infiltrates composed of lymphocytes and plasma cells. Additional review, including immunohistochemical staining for CD20, CD3, CD30, CD15, PAX5, MUM1, and CD68, showed no evidence of classical Hodgkin lymphoma. A core needle biopsy of a left supraclavicular lymph node (Figure 3) showed lymphoid tissue with a nodular architecture composed predominantly of small lymphocytes and macrophages. Scattered large pleomorphic cells with multilobulated nuclei and small nucleoli (“popcorn cells”) were identified and positive for CD20, CD79a, and PAX5. Background lymphoid tissue showed nodular aggregates of small B lymphocytes. There was a predominance of T lymphocytes expressing CD3, CD4, and PD-1. CD21 and CD23 highlighted the expansion of follicular dendritic cell networks. The biopsy confirmed NLPHL recurrence without histologic transformation.

**Figure 4:**
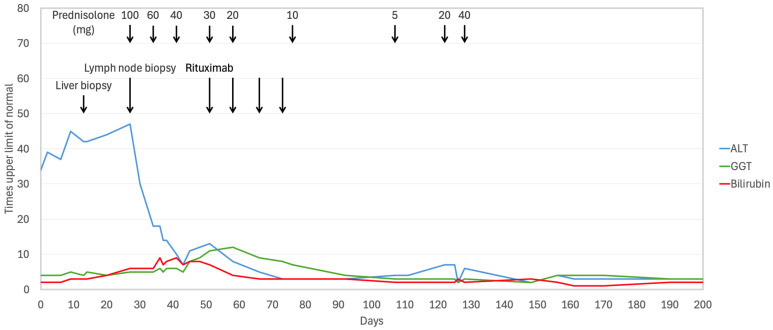
Course of liver function tests in relation to diagnostic and therapeutic interventions.

**Figure 5:**
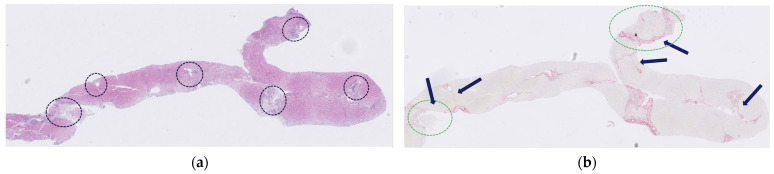
R Liver biopsy at diagnosis: (**a**) HE stain (12.5× objective lens equivalent). Portal tracts contain few inflammatory cells (black dotted circle); (**b**) collagen stain (Sirius Red) highlights bridging fibrosis (arrows), with features of nodularity (green dotted circle).

The concomitant relapse of NLPHL and the absence of an alternative etiology suggested that the hepatic dysfunction was secondary to paraneoplastic hepatitis. As LFTs further deteriorated, oral prednisolone was initiated at 100 mg/day, leading to rapid biochemical improvement. Prednisolone was tapered to 60 mg after one week and further to 40 mg the following week. The underlying lymphoma was treated with rituximab 375 mg/m^2^ weekly for four weeks, resulting in complete remission on positron emission tomography (PET) scan. By day 100, LFTs had decreased to less than five times the upper limit of normal (ULN), and prednisolone was further tapered to 5 mg daily. However, at this dose, laboratory results deteriorated again, which resulted in increased bilirubin (1.6 g/dL), ALT (328 U/L), and GGT (171 U/L). Prednisolone was increased to 20 mg and, subsequently, to 40 mg, resulting in partial improvement, though ALT levels never fully normalized (Figure 4). Sustained remission was confirmed by a repeat PET scan at six months after the end of treatment, and a bone marrow biopsy at nine months showed no evidence of lymphoma infiltration. Repeat work-up for liver disease was performed. Viral serology was negative for hepatitis A and E, human herpesvirus 8, and herpes simplex virus. Polymerase chain reaction for CMV and EBV was negative. Abdominal ultrasound showed a heterogeneous liver parenchyma with slightly irregular intrahepatic bile ducts, patent vasculature, and no focal lesions. Liver stiffness measurement was 10.8 kPa, consistent with severe fibrosis (F3). Magnetic resonance cholangiopancreatography (MRCP) showed irregular hepatic contours and extensive fibrotic changes predominantly involving the left hepatic lobe. The hepatic vasculature was patent, and no focal hepatic lesions were identified. The biliary tree and gallbladder appeared normal, with mild elongation of peripheral intrahepatic bile ducts without signs supporting a diagnosis of primary sclerosing cholangitis (PSC). There were no imaging signs of portal hypertension. The spleen was normal in size (11 cm). Repeat liver biopsy (Figure 5) demonstrated mild portal inflammation composed predominantly of lymphocytes and occasional plasma cells, without interface activity or bile duct injury. Collagen stain revealed perisinusoidal and bridging fibrosis. There was no evidence of steatosis, iron, or copper deposition.

**Figure 6:**
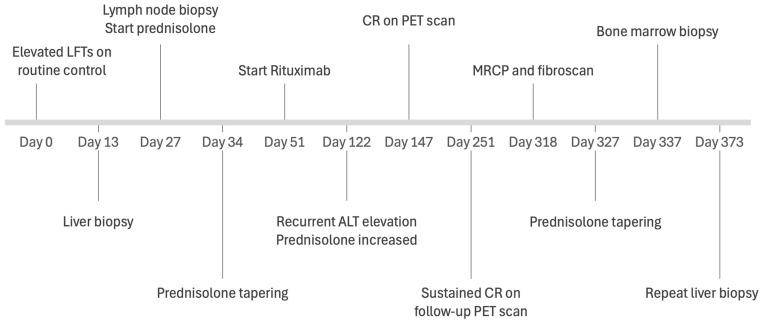
Clinical timeline of the case report showing time points of diagnostic evaluations, therapeutic interventions, and clinical responses.

Prednisolone tapering was continued. Potentially hepatotoxic medications such as alendronate, co-trimoxazole, and pantoprazole were stopped. Liver tests stabilized at a low but not normal level. Follow-up was carried out every six to eight weeks. The clinical timeline, including diagnostic evaluations, therapeutic interventions, and clinical responses, is summarized in Figure 6.

## 3. Discussion

PNS are defined as clinical manifestations caused by an underlying malignancy that are not attributable to compression or direct tumor invasion. We report a case of paraneoplastic hepatitis associated with relapsed NLPHL. The diagnosis was based on asymptomatic elevations in serum transaminases (up to 47 times the ULN) and a clear temporal association between hepatic injury and relapse of NLPHL. Histopathologic findings demonstrated lymphocytic infiltration of the liver without evidence of malignant hepatic involvement. Simultaneously, relapse of NLPHL was diagnosed on pathologic examination of a lymph node biopsy. Alternative causes of liver injury were systematically excluded, including viral infections, autoimmune hepatitis, congenital or vascular liver disease, and drug-induced hepatotoxicity. Immunosuppressive therapy and lymphoma-directed treatment resulted in a prompt and sustained response, with recurrence of transaminase elevations during rapid corticosteroid tapering, further supporting an immune-mediated paraneoplastic mechanism.

There has been one previous case report of paraneoplastic hepatitis in NLPHL [8]. The patient, a 28-year-old man with a history of stage IA NLPHL treated with radiotherapy, presented with acute severe hepatitis characterized by jaundice, coagulopathy, and hypoglycemia. As in the present case, extensive evaluation excluded viral, autoimmune, and metabolic causes, and liver biopsy demonstrated severe hepatocellular injury without evidence of lymphomatous infiltration. Both patients exhibited a similar pattern of hepatocellular damage occurring concurrently with NLPHL relapse. Despite these similarities, the clinical courses differed substantially. The patient described by Deacon et al. presented with impending acute liver failure but achieved complete biochemical recovery after combined corticosteroid and immunochemotherapy [8]. In contrast, our case presented a more indolent course, with marked transaminase elevations but preserved hepatic function and subsequent progression to hepatic fibrosis despite remission of the underlying lymphoma. These observations suggest that paraneoplastic hepatitis in NLPHL may manifest along a clinical spectrum ranging from acute, reversible hepatic failure to chronic, fibrosing hepatitis, potentially reflecting variations in the intensity and duration of the immune response.

The pathogenesis of paraneoplastic hepatitis remains incompletely understood. One proposed mechanism involves immune cross-reactivity between tumor antigens and hepatocellular epitopes, leading to immune-mediated hepatocellular damage. In addition, tumor secretion of cytokines may amplify inflammatory signaling and exacerbate hepatic necrosis [15]. NLPHL is characterized by preserved B-cell antigen expression and the presence of PD1-positive follicular T-helper cells in the tumor microenvironment [16]. These immunologic features may theoretically facilitate aberrant antigen presentation and immune activation. However, there is currently no direct evidence linking these mechanisms to paraneoplastic phenomena in NLPHL.

Management relies on treating the underlying malignancy and modulating the immune-mediated hepatic inflammation. Rituximab, a CD20-directed monoclonal antibody and cornerstone in the treatment of relapsed NLPHL, likely contributed to disease control in this patient. The combination of rituximab and prednisolone may have played a complementary role in alleviating immune-mediated hepatic injury. However, resolution of hepatic dysfunction was only partially achieved and remained corticosteroid dependent despite complete remission of the lymphoma. Steroid-sparing immunosuppressive therapies, such as azathioprine or mycophenolate mofetil, were considered. Given the rapid response to corticosteroids and subsequent stable, mild elevation of liver enzymes (2–3 times ULN) without evidence of progression, escalation to additional immunosuppressive therapy was ultimately deemed unnecessary. The differential diagnosis for these ongoing liver abnormalities included drug-induced liver injury, viral reactivation, and steroid-related metabolic injury. Hepatic involvement by lymphoma was considered unlikely, given the complete metabolic response in other disease sites. Our most plausible explanation was ongoing immune activation or incomplete control of the paraneoplastic process, possibly related to overly rapid corticosteroid tapering. Eventually, repeated liver biopsy excluded lymphoma and showed histological evolution towards bridging fibrosis with minimal residual inflammation. This clinical course shares features with autoimmune hepatitis, in which relapse after treatment withdrawal is frequent and long-term maintenance treatment is often required [17]. In one study, sustained remission was achieved in only 17% of patients treated for less than four years, compared with 67% in those treated longer [18]. Although autoimmune antibodies were not identified in our patient, histological findings demonstrated mixed inflammatory infiltrates composed of lymphocytes and plasma cells, resembling patterns observed in autoimmune hepatitis. This overlap likely reflects shared immune-mediated inflammatory mechanisms. It raises the possibility that, as in autoimmune liver disease, gradual tapering of immunosuppression and achieving sustained biochemical and histological remission may be important considerations when managing paraneoplastic hepatitis.

Paraneoplastic phenomena can lead to early recognition of otherwise clinically occult tumors but may also complicate disease course and contribute to morbidity. This case emphasizes the importance of excluding alternative causes of liver injury and supports the use of liver biopsy as a diagnostic tool in complex presentations. It underscores the need for careful coordination of immunosuppressive and lymphoma-directed therapy to optimize both hepatic and lymphoma-related outcomes.

## 4. Conclusions

Paraneoplastic hepatitis represents a rare but important differential diagnosis in patients with NLPHL presenting with unexplained hepatic injury. This case broadens the clinical spectrum of the disease by illustrating an insidious and persistent disease course, distinct from previously reported cases characterized by acute and reversible hepatic dysfunction. Early recognition, exclusion of alternative etiologies, and histopathological confirmation remain essential for diagnosis. Analogous to the management of autoimmune hepatitis, sustained hepatic remission may require prolonged immunomodulatory therapy. Given the limited number of reported cases, optimal treatment strategies and long-term hepatic outcomes remain poorly defined. Improved understanding of the immunologic mechanisms may help identify patients at risk and guide therapeutic approaches.

## Data Availability

The data that support the findings of this study are available from the corresponding author upon reasonable request.

## Abbreviations

The following abbreviations are used in this manuscript:

NLPHL	Nodular lymphocyte-predominant Hodgkin lymphoma
HL	Hodgkin lymphoma
ICC	International Consensus Classification
NLPBL	Nodular lymphocyte-predominant B-cell lymphoma
WHO	World Health Organization
cHL	Classical Hodgkin Lymphoma
LFT	Liver function test
PNS	Paraneoplastic syndrome
ABVD	Doxorubicin, bleomycin, vinblastine, and dacarbazine
CR	Complete remission
PR	Partial response
ALT	Alanine transaminase
AST	Aspartate transaminase
GGT	Gamma-glutamyl transferase
LDH	Lactate dehydrogenase
INR	International normalized ratio
HIV	Human immunodeficiency virus
CMV	Cytomegalovirus
EBV	Epstein–Barr virus
CT	Computed tomography
PET	Positron emission tomography
ULN	Upper limit of normal
MRCP	Magnetic resonance cholangiopancreatography
PSC	Primary sclerosing cholangitis
HE	Hematoxylin and eosin
